# Endoscopic repair of traumatic sphenoid sinus meningoencephalocele with cerebrospinal fluid rhinorrhea: a case report and literature review

**DOI:** 10.3389/fsurg.2025.1665062

**Published:** 2025-10-08

**Authors:** Jiarong Tan, Chengcheng Zuo, Feiying Guo, Shunli Tang

**Affiliations:** 1 Department of Otolaryngology, Head and Neck Surgery, Huzhou Central Hospital, Fifth School of Clinical Medicine of Zhejiang Chinese Medical University, Huzhou, China; 2 Department of Dermatology, Huzhou Central Hospital, Fifth School of Clinical Medicine of Zhejiang Chinese Medical University, Huzhou, China

**Keywords:** trauma, meningoencephalocele, cerebrospinal fluid rhinorrhea, endoscopic surgery, case report and literature review

## Abstract

Traumatic sphenoid sinus meningoencephalocele with cerebrospinal fluid (CSF) rhinorrhea is a clinically rare condition, typically presenting as trauma-induced clear watery discharge from the nasal cavity and nasopharynx. Surgery is the mainstay of treatment, including open and endoscopic procedures. Herein, we report a case of traumatic sphenoid sinus encephalocele with CSF rhinorrhea that was successfully treated using an endoscopic transmaxillary posterior wall approach, and reviewed the literature on endoscopic approaches for this condition. Our clinical experience indicates that an endoscopic transmaxillary approach may be a novel and promising surgical option for this condition.

## Introduction

Meningoencephalocele with cerebrospinal fluid (CSF) leakage is a rare disease characterized by herniation of the dura mater, CSF, and often cerebral tissue through skull base defects, accompanied by abnormal CSF leakage from the intracranial space into the skull base or along the spinal column. If left untreated, it can lead to serious complications such as intracranial infections. Surgery is the mainstay of treatment, and can be classified into open or endoscopic procedures. Notably, endoscopic endonasal approaches have become widespread because they are minimally invasive and safe and lead to fewer complications.

Herein, we introduce a case of traumatic sphenoid sinus meningoencephalocele with CSF rhinorrhea that was successfully treated using an endoscopic endonasal transmaxillary posterior wall approach.

## Case presentation

**Figure 1:**
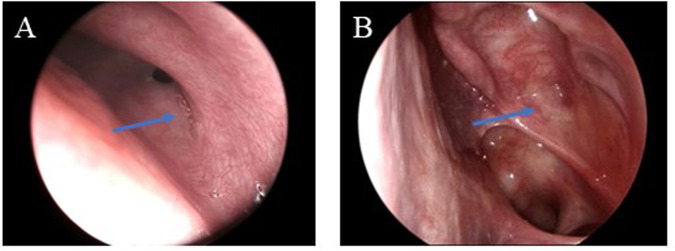
**(A)** Endoscopic nasopharyngoscopy shows left nasal watery discharge without other symptoms. The arrow indicates a little clear, watery discharge emanating from the sphenoid sinus ostium. **(B)** Postoperative endoscopic follow-up at 6 months demonstrates no evidence of CSF rhinorrhea and a successful reconstruction of the traumatic defect. The arrow indicates the satisfactory healing of the surgical repair site.

**Figure 2:**
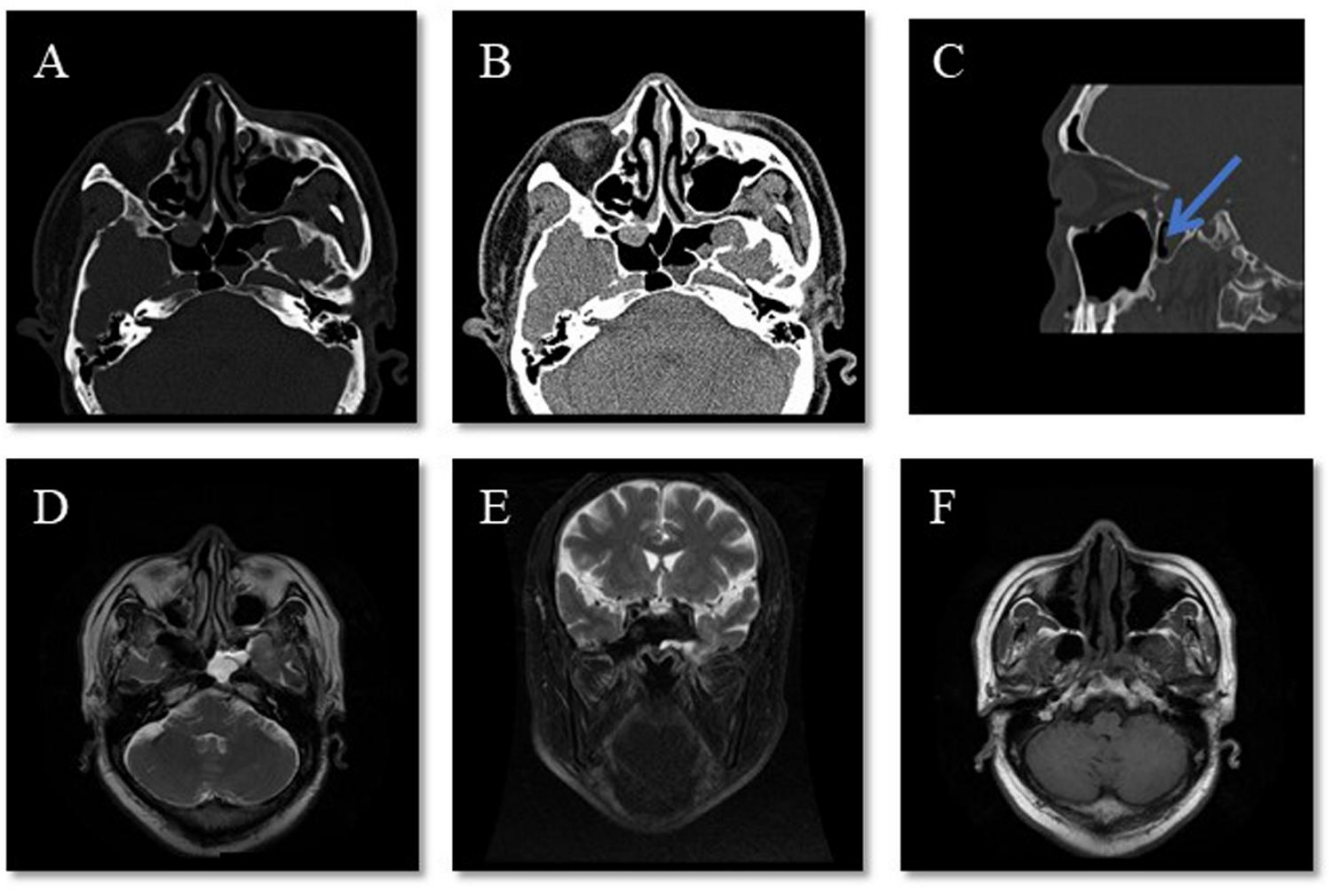
Representative radiographical images upon admission. **(A**–**C)** CT of the patient’s sinuses shows a continuity disruption in the lateral wall of the left sphenoid sinus and meningoencephalocele in the sinuses. The arrow indicates direct apposition between the sphenoid sinus recess and the posterior maxillary wall without intervening vascular structures. **(D**–**F)** MRI of the sinuses and brain shows effusion in the left sphenoid sinus and an abnormal cystic signal, which raised suspicion of it being brain tissue.

An otherwise healthy 60-year-old man presented to the Otolaryngology Department with a 6-month history of clear watery discharge from the left nostril following trauma. He denied any symptoms at the time of impact, but developed intermittent non-mucoid rhinorrhea from the left nostril 2 weeks later. Upon admission, the general physical examination, including the patient’s respiratory, cardiovascular, abdominal, and neurological systems, was unremarkable. The nasal examination revealed a normal appearance, and endoscopic nasopharyngoscopy showed watery discharge from his left nasal cavity without mucosal edema, congestion, or other symptoms (Figure 1A). An analysis of the nasal discharge revealed elevated glucose levels (6.78 mmol/L) and chlorine levels (183.7 mmol/L), suggesting it was CSF. Radiological investigations, including computed tomography of the patient’s sinuses (Figures 2A–C) and magnetic resonance imaging of his sinuses and brain (Figures 2D–F) showed a continuous disruption in the lateral wall of the left sphenoid sinus, leading to CSF leakage, and a suspicion of herniated brain tissue in the left sphenoid sinus, namely meningoencephalocele. The patient was preoperatively diagnosed with traumatic sphenoid sinus meningoencephalocele with CSF rhinorrhea.

**Figure 3:**
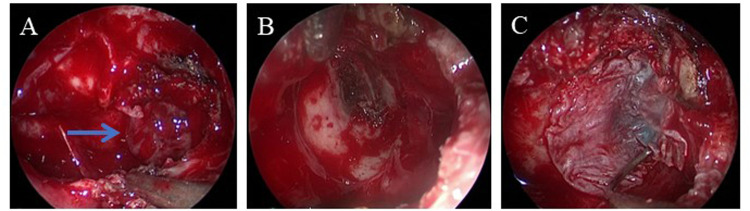
Intraoperative images. **(A)** Herniated meningeal and brain tissues in the left sphenoid sinus. **(B)** After removing the herniated tissues, a bone defect in the left lateral recess of the sphenoid sinus is found, together with clear liquid outflow. **(C)** Muscle and fascia lata from the lateral thigh are used to repair the defect. The arrow indicates the herniated meningeal and brain tissues.

An endoscopic nasal repair was performed using a transmaxillary posterior wall approach after obtaining preoperative informed consent and ascertaining the patient's fitness for anesthesia. During the surgery, the anterior wall of the sphenoid sinus was removed and the maxillary sinus ostium was widened. Posterior dissection was carried out along the maxillary sinus toward the root of the pterygoid process. The bony structures of the pterygoid process root and the posterior wall of the maxillary sinus were then removed. A bone defect was observed in the left lateral recess of the sphenoid sinus, with herniation of meningeal and brain tissues, and visible CSF leakage (Figure 3). After removing the herniated brain tissue and CSF, a multilayered repair was performed using the inside-out technique, i.e., using muscle and fascia lata from the lateral thigh, gelatin sponge, and iodoform gauze to close the defect. Postoperatively, the patient was treated daily with 20% mannitol to reduce intracranial pressure and ceftriaxone to prevent infection for 12 days. The iodoform gauze in his left nasal cavity was removed 10 days after the surgery. The patient was then discharged without a nasal drip, fever, headache, or any other complications. The postoperative follow-ups at 1 and 6 months confirmed the absence of CSF rhinorrhea and demonstrated successful reconstruction of the traumatic defect (Figure 1B).

## Discussion

Meningoencephalocele is a rare skull base defect characterized by the herniation of dura mater, CSF, and often cerebral tissue. It is etiologically classified into three types, namely congenital, spontaneous, and traumatic types, with a ratio of approximately 5:4:1 (1). Depending on the anatomical site of herniation, meningoencephalocele is also categorized into sincipital, basal, and retrooccipital types, with a ratio of approximately 3:2:15 (2–4). Patients usually present with a series of symptoms, including headaches, seizures, and signs of meningitis.

CSF leakage is an uncommon but significant condition characterized by egress of CSF from the intracranial cavity through abnormal communications between the subarachnoid space and pneumatized structures within the skull base, typically the sinonasal tract, middle ear, or mastoid system (5, 6). CSF rhinorrhea refers to abnormal CSF leakage from the intracranial space into the nasal cavity or nasopharynx, most commonly into the anterior middle fossa and sella turcica. The clinical features include unilateral clear watery drainage, positional or exertional clear rhinorrhea, poor response to rhinitis medication, and a history of trauma or surgery (7). Patients may also present with non-specific symptoms such as headache, dizziness, anosmia or ageusia, pulsatile tinnitus, and hearing impairment (1).

Meningoencephalocele is strongly associated with CSF leakage, with a reported frequency ranging from 50% to 100% (1). Previous studies reported that head injuries are the primary cause of adult CSF leakage, accounting for 80%–90%. Moreover, 10%–40% of adult skull base fractures are accompanied by CSF leakage (1, 3). CSF leakage may progress to meningitis, with an annual rate of approximately 10%, which rises to 40% in patients with non-obvious trauma-induced CSF leakage and repeated intracranial infections. CSF leakage can provide diagnostic clues for meningoencephalocele. Notably, the differential diagnoses for CSF leakage include other rhinorrhea diseases, namely, vasomotor rhinitis, sinus cysts, secretory otitis media, and other conditions (6). A delayed diagnosis may pose considerable risks and lead to serious complications, such as meningitis, encephalitis, brain abscess, epilepsy, or death.

Surgery is the primary therapy for traumatic CSF leakage with or without meningoencephalocele and is generally performed 2 weeks after trauma (5). Surgical approaches are classified into open and endoscopic procedures. Owing to the lower overall success rate and high complication rate of open surgery, endoscopic endonasal approaches are gradually becoming more common (5, 8, 9). Depending on the preoperative imaging of defect sites, multiple endoscopic endonasal approaches have been reported, including the single-nostril and/or double-nostril techniques, the transnasal sphenoid approach, the transnasal maxillary approach, the middle cranial fossa approach, and the lateral nasal process approach. Septal flaps, middle turbinate flaps, cartilage, fascia lata, and fat are commonly used as restorative materials in endoscopic procedures.

In this case, considering the extensive lesion accompanied by brain tissue herniation from the middle cranial fossa, the transsphenoid/transpterygoid approaches would provide insufficient operative space. Thus, a transmaxillary posterior wall approach was employed. This procedure was considered safe because it allowed adequate exposure of the far lateral sphenoid sinus lesions, exposed the lateral recess of the sphenoid sinus, and preserved the sphenopalatine artery. Preoperative imaging in this patient demonstrated that the sphenoethmoidal recess was located adjacent to the posterior wall of the maxillary sinus. The absence of vascular structures in this region further supported the safety of this surgical approach. Using lateral myofascial thigh tissue as the restorative material rather than fat grafts may help avoid postoperative fat resorption-induced headaches (1). Iodoform gauze on the outermost layer reduces seepage, promotes local granulation tissue growth, and prevents adhesion.

Sphenoid sinus meningoencephalocele with CSF rhinorrhea is rare in clinical practice and difficult to diagnose because rhinoscopy cannot easily detect intrasphenoidal meningoencephaloceles, and CSF leakage in such cases often presents with atypical symptoms. We also reviewed the literature on traumatic sphenoid sinus meningoencephalocele with CSF rhinorrhea and the findings in Table 1. As of January 2025, six identifiable patients with traumatic sphenoid sinus meningoencephalocele and CSF rhinorrhea have been reported. All the patients were successfully treated using endoscopic repair without noticeable postoperative infections and complications, including three patients who experienced previous failure of surgical reconstruction. All the patients (including ours) presented with mild symptoms, leading to missed or delayed diagnoses, as previously described. Unlike prior cases that used binostril and mononostril transpterygoid approaches, we employed an endoscopic endonasal transmaxillary posterior wall approach in our patient without obvious postoperative complications, suggesting that this may be a novel and promising surgical method for treating traumatic sphenoid sinus meningoencephalocele with CSF rhinorrhea.

**Table 1:** Details of studies on traumatic sphenoid sinus meningoencephalocele with cerebrospinal fluid rhinorrhea.

Reference	Age	Gender	Symptom duration	Other symptoms	Previous treatment	Surgical procedure	Reconstruction	Follow-up
Ulu et al. (8)	14	Male	4 weeks	No	Transcranial surgery	Endoscopic endonasal mononostril transpterygoid approach	Pedicled nasoseptal flap	Recovered without recurrence
Isler et al. (5)	41	Male	NA	No	Transcranial surgery, endoscopic endonasal surgery	Endoscopic binostril endonasal approach	Abdominal fat, fascia lata	Recovered without recurrence
	46	Male	NA	No	Transcranial surgery	Endoscopic binostril endonasal approach	Abdominal fat, abdominal fascia, nasoseptal flap	Recovered without recurrence
	39	Female	NA	No	No	Endoscopic binostril endonasal approach	Abdominal fat, abdominal fascia, abdominal muscle, middle turbinate, pedicled nasoseptal flap	Recovered without recurrence
Xue et al. (9)	56	Male	NA	Headache, hyposmia	Nasal polyp surgery	Endoscopic endonasal mononostril transpterygoid approach	NA	Recovered without recurrence
	41	Female	NA	No	No	Endoscopic endonasal mononostril transpterygoid approach	NA	Recovered without recurrence

Studies on traumatic meningoencephalocele with cerebrospinal fluid leakage that lacked identifiable patient information were not included in the table.

In summary, an endoscopic endonasal transmaxillary posterior wall approach appears to be an effective therapy for repairing traumatic sphenoidal encephalocele with CSF rhinorrhea. However, its efficacy and safety require further exploration and validation.

## Conclusion

Traumatic sphenoid sinus meningoencephalocele with CSF rhinorrhea is a rare condition that requires careful diagnosis. An endoscopic endonasal transmaxillary posterior wall approach is a promising reconstructive technique for this condition, especially in cases with extensive lesions and avascular courses posterior to the maxillary sinus. Further studies with large sample sizes are necessary to confirm the efficacy and safety of this approach and to elucidate the potential preoperative laboratory or imaging characteristics that may suggest optimal indications.

## Data Availability

The raw data supporting the conclusions of this article will be made available by the authors, without undue reservation.
